# Navigating cancer using online communities: a grounded theory of survivor and family experiences

**DOI:** 10.1007/s11764-017-0616-1

**Published:** 2017-05-03

**Authors:** Lydia Jo Harkin, Kinta Beaver, Paola Dey, Kartina Choong

**Affiliations:** 1 Division of Psychology, Nottingham Trent University, Nottingham, Nottinghamshire NG4 1BU UK; 2 School of Health Sciences, University of Central Lancashire, Preston, Lancashire PR1 2HE UK; 3 Faculty of Health and Social Care, Edge Hill University, Ormskirk, Lancashire L39 4QP UK; 4 Lancashire Law School, University of Central Lancashire, Preston, Lancashire PR1 2HE UK

**Keywords:** Cancer, Online support, Supportive care, Social media, Qualitative research, Grounded theory

## Abstract

**Purpose:**

People affected by cancer often have unmet emotional and social support needs. Online cancer communities are a convenient channel for connecting cancer survivors, allowing them to support one another. However, it is unclear whether online community use makes a meaningful contribution to cancer survivorship, as little previous research has examined the experience of using contemporary cancer communities. We aimed to explore the experiences of visitors to online cancer communities.

**Methods:**

Twenty-three in-depth interviews were conducted with online cancer community visitors, including cancer survivors (*n* = 18), family members (*n* = 2), and individuals who were both a survivor and family member (*n* = 3). Interviews were analysed using a grounded theory approach.

**Results:**

A theory developed explaining how individuals ‘navigated’ the experience of cancer using online cancer communities. Online advice and information led participants on a ‘journey to become informed’. Online friendships normalised survivorship and cast participants on a ‘journey to recreate identity’. Participants navigated a ‘journey through different worlds’ as they discovered relevant and hidden communities.

**Conclusions:**

This theory highlights virtual paths people affected by cancer can take to self-manage their experience of the disease. Online community experiences can be improved by promoting online evaluation skills and signposting visitors to bereavement support.

**Implications for cancer survivors:**

Cancer survivors can benefit through both lurking and posting in online communities. However, individuals risk becoming distressed when they befriend individuals who may soon die. Additionally, people affected by rarer cancers can struggle to find shared experiences online and may need to look elsewhere for support.

## Introduction

By 2030, there will be approximately 23.6 million new cases of cancer reported worldwide each year [1]. Cancer survivorship can have significant negative psycho-social consequences. Survivors and their families can both struggle to adjust to their fear of the cancer survivor dying, uncertainty of the outcome of treatment, and perceived lack of control over the future [2–4]. Moreover, reviews have estimated that approximately 20–30% of cancer survivors and spouses experience depression and anxiety after a cancer diagnosis [5, 6]. Consequently, clinical cancer care guidelines in the UK and USA have recommended that individuals be offered social and emotional support to alleviate feelings of distress [7, 8]. However, as a major worldwide chronic disease, providing psycho-social support for the growing cancer survivor population is also a significant economic burden [9, 10]. Thus, there has been a call for more affordable and efficient ways of offering support to people and families affected by cancer [10].

Peer support is considered a relatively effective way of providing social and emotional support during cancer survivorship [11, 12]. Individuals have gained unique insights into the illness experience by communicating with fellow patients or families [13, 14]. Information shared between peers includes advice on coping, gained through the lived experience of cancer [13]. Therefore, peers are well placed to support one another [13]. Accordingly, people living with cancer and their families have been encouraged to attend local cancer support groups, to meet and share support [7]. However, face-to-face support groups have typically reported low rates of attendance, with high drop-out rates [13]. The practicalities involved in attending face-to-face support groups mean that they may not be convenient for people affected by a life-limiting illness. Support groups are often scheduled infrequently at inappropriate times for people undergoing active cancer treatment or at inconvenient times for individuals with demanding caregiver responsibilities [14]. In addition, attending a physical support group poses challenges for individuals who may be unable to drive, with poor transport links, or who live in isolated rural areas [12]. Thus, more accessible peer support has the potential to offset the social isolation commonly experienced by cancer survivors.

The internet offers a convenient way to connect people affected by cancer. Online communication allows individuals to support one another without the challenges of physically attending support groups, and from the comfort of home. In the European Union (EU), Great Britain, and the USA, 81, 87, and 78% of households respectively have access to the internet [15–17]. The rise of mobile and tablet technology has also increased access to online networks and communication [18]. Figures suggest that nearly two thirds of American adults [19] and approximately 290 million Europeans use social media sites to communicate online [16]. Furthermore, internet and social media use is becoming increasingly entrenched in health behaviours. Amongst a sample of cancer survivors, a French study recorded 85% of people regularly participating in online activities such as online health communication [20]. Moreover, studies categorising cancer-related information online have reported increasing numbers and varieties of ‘online cancer communities’ [21, 22]. These online spaces are dedicated to hosting conversations between cancer survivors and their families. Consequently, evidence suggests that internet use is pervading western lives and that many people affected by cancer could have the opportunity to meet and share their experiences online.

Online communities enable visitors to send messages to fellow survivors by posting questions, answers, information, and resources to a shared space. Studies have analysed the messages in online cancer communities and found that they regularly contain expressions which could support individuals’ self-esteem and could meet needs for information and emotional support [23–25]. Cancer communities have offered emotional support in the form of empathetic statements to others, displays of warmth, and in virtual offers of physical affection such as hugs [24]. In communities for families affected by cancer, members have encouraged efforts in caring and managing the illness, leading to enhanced self-esteem [25]. Such studies have framed online cancer communities as resources which could offer supportive benefits for cancer survivors and their families.

Some clinicians and academics have expressed concern that online cancer communities may not be a valuable source of support or could even be harmful to vulnerable cancer survivors [26]. Studies have found that the majority of visitors do not post messages to online communities but simply read messages in communities (also known as ‘lurking’) [27]. This significance of lurking has remained unclear, and studies have highlighted that it is unknown whether lurking has a greater or lesser emotional impact on cancer survivorship compared with posting [28]. Furthermore, people affected by cancer commonly experience a wide range of emotional sequelae, including worry and distress [6]. These fears and uncertainties have been expressed with high prevalence within online communities [29, 30]. Reading distressing and fearful messages could exacerbate individuals’ negative experiences of cancer [29]. Many online communities do not have the presence of a trained specialist to support the complex needs of people living with and beyond cancer [21]. Additionally, communities are not commonly monitored for accurate information, and therefore, false information and rumours could be shared in the communities [29]. This could lead people to have false expectations concerning the illness, disappointment with their treatment progression, and renewed feelings of uncertainty and distress regarding cancer.

Despite the potential for support in online cancer communities, there is relatively limited understanding of how these groups impact the experience of cancer survivorship [31]. Many studies have highlighted the content of cancer community messages posted online [31]. However, there has been a dearth of evidence regarding how people affected by cancer experience such online communities and how visiting can influence life after a cancer diagnosis. A more holistic insight is required before online cancer communities can be understood as a resource for psycho-social support. In this context, a grounded theory is warranted in order to elicit a rich understanding of online community interactions in the context of the cancer survivor and their families’ multifaceted experiences of cancer. To the best of our knowledge, grounded theory had not previously been employed in the understanding of online health communities. Moreover, due to the burgeoning development of online communication, no theory has been used to explain how online supportive communication can impact cancer survivors and their families. However, a theoretical guide developed through grounded theory would support the progress and development of future online cancer support. This study aimed to explore the experiences of visitors to online cancer communities and to generate a grounded theory of online cancer community use amongst people affected by cancer.

## Methods

### Study design

This was a qualitative interview study with people who had visited online cancer communities and who self-identified as ‘affected by cancer’ because they had been diagnosed with cancer or were family of those who had been diagnosed. Qualitative research has been recommended for areas in which experiences are little known, as these methods facilitate in-depth exploration of people’s interactions and perceptions of a phenomenon [32]. Constructivist grounded theory was used to guide the interviews and analysis [33]. Grounded theory allows the researchers to be sensitive to patterns across people’s experiences [33]. This approach facilitated the development of theory. Furthermore, the well-cited constructivist form of grounded theory incorporated a critical appraisal of how the researchers shaped interviews, data interpretation, and the presentation of the findings [33].

### Recruitment

To elicit a nuanced view of digital communities, we decided to collect data from a range of individuals including current patients, long-term cancer survivors, and family of cancer survivors. Similarly, cancer populations affected by different cancer types, at different stages in the cancer trajectory, and undergoing different clinical treatments have been found to interact with one another within online cancer communities [24]. Thus, no limits were placed on the diagnostic stage or the type of cancer that participants were experiencing. In addition, there has been no consistent and accepted definition of an ‘online cancer community’, nor a definition of what level of activity constitutes online community engagement. Therefore, we simply stipulated that participants had accessed communities at least once. We did not place parameters on what online group the population had used. Participants were deemed eligible if they were aged 18 and over, as this study did not explore the experiences of children cancer survivors.

An advertisement calling for participation was sent to 19 organisations based in the UK offering online and offline psycho-social support for a range of different cancer diagnoses. The advertisement described the study and encouraged anyone who believed they were eligible to contact LJH. When they made this contact, individuals received study information sheets which assured ethical compliance, such as anonymity and the use of anonymised data in publications. Individuals were also assessed to ensure they had visited online cancer communities, and information about their age, gender, how they had been affected by cancer, and the type of online communities they had visited was recorded for later theoretical sampling. The sample of participants was drawn from individuals who confirmed their consent to take part by returning consent forms electronically or by post. Initially, all individuals were invited for interview. As themes emerged from data analysis, participants were selected according to their ability to contribute new insights into the data, otherwise known as theoretical sampling [33].

### Data collection

Participants were offered a choice of interview settings: face-to-face, by telephone, or by the video calling software Skype. The interviews were semi-structured, using an interview guide (see Appendix Table 2, interview schedule) which was reviewed by a lay and carer advisory board for relevance and clarity. Interviews were conducted by LJH, who had received training in interviewing and sensitive communication skills.

### Data analysis

Interviews were audio recorded and transcribed verbatim. QSR-NVivo was used to store and manage the data. The data were analysed using a constant comparison approach in which LJH familiarised herself with and analysed transcripts between each interview. Initially, the data analysis involved creating descriptive, line-by-line codes for each transcript. As findings emerged, codes were combined to produce themes which reflected key experiences in the data. This analysis process followed recommended guidelines for constructivist grounded theory [33]. Analysis was primarily conducted by LJH, and at monthly intervals, the analysis was presented to three team members (KB, PD, and KC) alongside original transcripts, to appraise the developing codes and themes with the original data.

### Ethics

Informed consent was obtained from all individual participants included in the study. To assure participants’ confidentiality and anonymity, all identifying information was removed from the transcripts, and participants were referred to according to identification codes (see Box 1). In addition, the specific online communities that were used by participants have not been named in this publication.

**Table:** 

Box 1: Participant identification codes
e.g. (P1^a^/F^b^/sarcoma^c^/survivor^d^)
^a^Participant number
^b^Participant gender
^c^Type or location of cancer
^d^Relationship to cancer

This research study obtained ethical approval from the University of Central Lancashire’s Science, Technology, Engineering, Medicine and Health Ethics Committee.

## Results

### Participants

Thirty-eight individuals responded to the study advertisement. However, 15 individuals decided that they did not wish or were unable to participate in the interviews. Twenty-three individuals were interviewed: 17 by telephone, 4 face-to-face, and 2 using Skype. Approximately 27 h of audio recorded data was captured, with interviews lasting an average of 69 min, with a range of 43–123 min.

Participants had been affected by a range of different cancer types. The most common diagnoses were melanoma and breast and ovarian cancers. The majority of participants accessed online cancer communities because they were living with a personal cancer diagnosis, though the sample also included participants who used online communities as a family member affected by cancer and individuals affected by both their own and a family member’s cancer. Table 1 summarises the characteristics of the study sample.

**Table 1:** Characteristics of the study sample

Participant demographics	Number
Relationship to cancer	
Cancer survivor	18
Family member	2
Both cancer survivor and family member	3
Cancer location/type	
Skin	7
Ovary	6
Breast	5
Bowel	2
Prostate	2
Brain	1
Head and neck	1
Lung	1
Non-Hodgkin’s lymphoma	1
Pancreas	1
Sarcoma	1
Thyroid	1
Age (years)	
<31	0
31–40	4
41–50	8
51–60	5
61–70	5
70>	1
Gender	
Female	19
Male	4
Ethnicity	
White British	22
Other	1

### The substantive grounded theory: navigating cancer using online communities

**Fig. 1:**
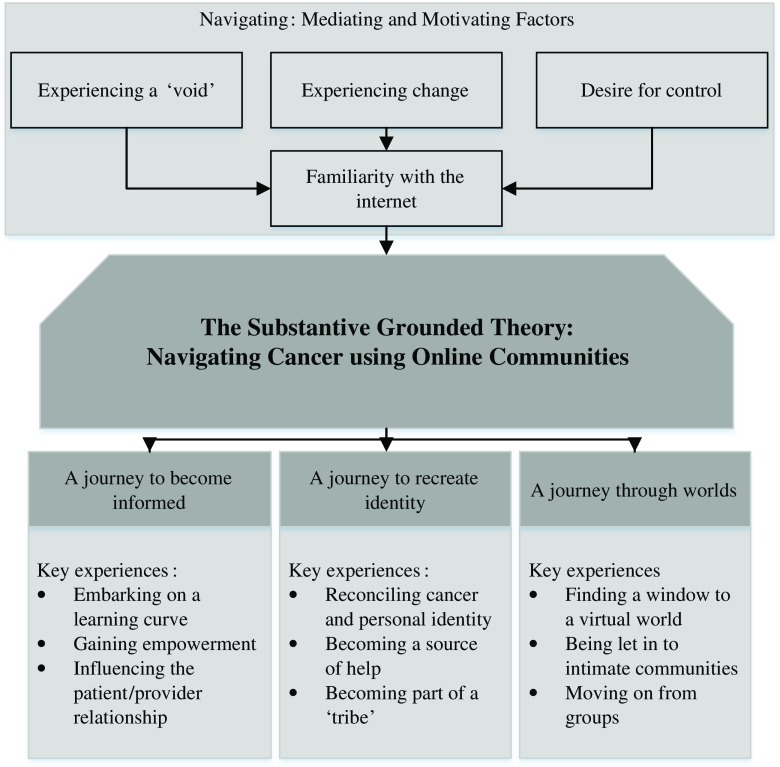
Navigating cancer using online communities

This study developed the substantive theory ‘navigating cancer using online cancer communities’ to explain how online communities influenced the experience of living with cancer (see Fig. 1). The title of this theory was drawn directly from participant descriptions. For the majority of the participants, the cancer diagnosis had a profound emotional impact on their lives, causing a range of practical challenges from diagnosis through long-term survivorship. Online communities were largely perceived as a rich resource of information and support. They enabled individuals to chart their progress through clinical procedures, to move past emotionally charged experiences, and to map out what they could expect to encounter in the future. Moreover, online communities were a unique resource to participants because they could be considered separate from face-to-face encounters with friends, family, and healthcare professionals. Participants were able to log in without informing their family members or healthcare professionals. This often allowed participants to express their feelings about the cancer experience without causing upset to their friends and family. In addition, the communities were a source of support accessible day and night, when traditional sources of cancer support such as families, telephone support lines, and healthcare professionals were unavailable. Several participants, nevertheless, found limited personal value in communities, as they did not feel the need for additional supportive resources beyond what was available to them through healthcare professionals, friends, and family. Thus, this substantive theory represented online community experiences for individuals who expressly wished for support in navigating the cancer experience.

*‘Interviewer: why were the communities so important to you? Participant: to work out, to try and navigate our way through. Because you have to make a lot of quite big choices in a short period of time. And I think you are probably quite ill equipped to do so…’ *(P17/F/ovarian/survivor)

The substantive theory had four main elements. These elements were often experienced concurrently and sometimes highlighted competing and conflicting requirements of online communities. For clarity, each element has been described distinctly in this paper. Primarily, participants described the experience of navigating and detailed what motivated and mediated this navigation when they used online cancer communities. Participants also felt that navigation led them on three main journeys in their cancer experience. These have been outlined in this theory as ‘a journey to become informed’, ‘a journey to recreate identity’, and ‘a journey through different worlds’.

### Navigation: motivating and mediating factors to using online communities

Navigating was a process that drove and directed participants’ use of online communities. Participants were focused on moving away from negative experiences they encountered during their cancer pathway or when caring for their family member. One key experience that motivated navigating cancer was *experiencing a void* after receiving the initial cancer diagnosis and during healthcare consultations. Participants were often unable to absorb and retain the information they received from healthcare professionals. Participants attributed this to feeling highly distressed and being unfamiliar with the complex medical information associated with the diagnosis and treatments. Thus, they perceived a void or an absence in knowledge which could allow them to cope with the illness. Online communities provided answers to questions that cancer survivors and family members had not asked or had forgotten to ask during consultations with healthcare professionals. The webpages could be browsed at leisure, and participants could spend hours deciphering the meaning of medical terms and procedures.

*‘You think ‘okay where do I go from here’ and this whole platform opens up of… like this void that you’ve never, this world opens up… I don’t know what the hell I’m doing, I don’t know what I’m up against, I don’t know what it means so, that was the initial reaction. I couldn’t wait to go online.*’ (P5/F/melanoma/survivor)

A second key motivation for navigating online communities was participants’ struggle with *experiencing change* in their lives as a result of the diagnosis. Daily life suddenly centred on healthcare appointments, therapeutic treatments, and other aspects of illness. This contributed to a feeling that cancer had caused participants’ lives to be abruptly altered. Most participants also felt that their existing social network struggled to understand this new altered life, as their friends and family had limited experiences of living with cancer. Online communities contained groups of people who were willing to offer support and guidance about how they had adapted to cancer, and this practical advice was welcomed by many participants in this study. As such, participants came to understand online communities as a space to ‘vent’ or communicate about aspects of their lives that had changed due to the cancer diagnosis.

*‘One of the reasons that I did sort of start looking at the internet support groups was because I tend to not cry in front of my husband, because he would get upset. And my friends did not really understand because as much as they were there for me, none of them had been through it.’ *(P15/F/breast/thyroid/both family and survivor)

A third motivation for participants using communities to navigate cancer was their *desire for greater control* over what they perceived as the ‘chaos’ of the diagnosis. People living with cancer believed that the key to finding order and control was understanding the healthcare procedures they were undergoing and that they may face in the future. Similarly, families sought to understand what their family member with cancer was experiencing, and predict what they would likely experience, so they could plan for their future. Online communities allowed participants to regain control because of the depth and detail of discussions that took place online. Several participants used these discussions to draw up lists of what they might experience and how to react if, or when, they also encountered those experiences.

*‘It [using online communities] did make an impact because I felt I was sort of more in control. Rather than everything just happening and me having to react to it, I could plan for things and think about them and think of the best way.’* (P10/F/pancreatic/family)

Most participants highlighted that their use of online communities was mediated and influenced by their *familiarity with the internet* in their everyday lives. Participants believed that there was nothing unusual in referring to the internet in the face of a life-threatening illness. Internet technology was an acceptable resource for participants. All participants were regular internet users, and many, but not all, had used social media in their lives before cancer.

*‘I just think it’s anything that a normal woman would do in that situation.’* (P7/F/ovarian/survivor)

### A journey to become informed

Communities allowed most participants to move from being in a position of limited knowledge to becoming better informed about cancer. Becoming informed through online communities was described as *embarking on a learning curve* because the tasks of understanding community messages and interacting with other group members were difficult for all participants. Most participants believed that a large body of important information existed in online communities but it was a steep and demanding task to acquire this complex knowledge. Participants’ knowledge base needed to be built from small bite-sized pieces of information and simple facts accumulating to knowledge on more complex topics. Participants also needed to learn how to distinguish relevant from irrelevant information online. Several participants were unable to do this and became anxious, scared, and prepared for outcomes in cancer treatment and prognosis that were ultimately irrelevant to their personal experiences.

‘*There’s all words and new phrases, that a year ago, I would not have known what my neutropenia reading would be and all these different things. And what the CA125 is. It’s quite a learning curve*.’ (P13/F/ovarian/survivor)

*‘I think the one thing I’d sort of, you know, go back and speak to myself five months ago, six months ago, before I started looking in the forums is … not to try to find every single answer and work out every single scenario by going on and looking at what other people have said.’* (P15/F/breast/thyroid/both family and survivor)

Several participants found that they were *gaining empowerment* as they became better informed. Participants associated becoming empowered with the ability to be an agent in their own experience of cancer. The information in communities encouraged individuals to take an active role in treatment decision-making. Moreover, online communities encouraged and affirmed participants’ practices of self-care. For instance, most participants valued discussions which included many different tips for coping, even if the tips were contradictory. Participants selected the advice that best suited their needs and preferences. In this trial and error fashion, individuals developed a sense of what best suited their personal needs.

*‘You could share that online with people about ways you had coped and what you’d achieved. And the response was always very, very supportive. People would say you know ‘that’s really good’, ‘well done’ and things like that… I felt it was very, confidence building.’* (P4/F/non-Hodgkin lymphoma/survivor)

Information from online communities *influenced the patient/provider relationship* for many participants in this study. Many participants were acutely aware of the precious and limited time they had available for discussion in healthcare appointments. Most participants believed that if they already had a foundation of knowledge of concepts that were being discussed by healthcare professionals, they could have greater participation in consultations. Online communities provided the means to discover this foundational knowledge. Community members shared their experiences of healthcare professional interactions and highlighted important information that had been useful to them when attending consultations.

*‘If I’m already understanding what they’re going to say and some of the terminology, that’s helpful… I feel like I’m in a better place to ask questions, or they don’t have to maybe waste time repeating stuff that I already know.*’ (P3/F/mal. melanoma/survivor)

Unfortunately, not all participants were able to find answers to their questions online. In particular, individuals living with rare and little understood cancer diagnoses could not find in-depth information relevant to themselves. One woman with a rare form of ovarian cancer highlighted that this was disappointing and caused her to feel isolated. This was a trigger for some individuals to reduce the amount of time they spent using online communities.

*‘There are questions that I wanted the answers to. What about the non-invasive implants that I’ve got? Has anyone got any experience of these? And what’s happened? Has anybody died from this? And it’s all little questions that I wanted the answers to that I feel like I never got.’* (P7/F/ovarian/survivor)

### A journey to recreate identity

Participants used online communities to address the disparity they felt between their identity before cancer and their lives following the cancer diagnosis. For participants living with cancer, this journey often involved making sense of a new self with cancer and moving on to recreate a vision of their future. For families affected by cancer, this involved understanding how the roles they had previously had, such as spouse, son, or sister, might change in light of the cancer diagnosis.

*‘I started using the social media to try and work out, to try and make sense of my own feelings.’* (P17/F/ovarian/survivor)

‘That was always my job as the, I was always the twin that perked her [sister with cancer] up when she was low, you know, right from being babies. I could make her laugh… And that was just my job, so the forum helped me do that, keep her smiling.’ (P14/F/brain/family)

Online communities helped most participants to *reconcile cancer and personal identity*. Participants needed to learn what it meant to be affected by cancer. They also needed to establish how being affected by cancer would dictate their future and change aspects of their lives that had been important to them. In addition, fellow community members were able to reassure participants that their lives were not entirely negative, despite the effects of cancer. Thus, experiential information was a valuable feature of online communities and essential for participants who questioned their identity. Other community members’ experiences helped to normalise the experience of cancer and shaped perceptions of their new identity with cancer.

‘[forum name] helped me reconcile myself to the fact that I was now retired. I might be in recovery but I was retired… it helped me learn to live with it.’ (P20/M/head and neck/survivor)

*‘I did find it useful to kind of read about other people’s experiential knowledge and how their, sort of, journey through had worked out.*’ (P17/F/ovarian/survivor)

Participants could take on a helping role in online communities, and most participants utilised this feature to different degrees. This involved providing assistance and support to other community members. Individuals answered the questions of other members and posted any items that might interest the community including pictures, information, or signposting to other websites. Most participants reasoned that *becoming a source of help* was driven by their gratitude after benefitting from the groups. Furthermore, helping others online gave participants a sense of pride and well-being. Several participants also wanted to give back to charities that had supported them or their family and so provided support in their online forums.

*‘So when there’s like new ladies coming along that are just going through diagnosis or first round chemo. That’s where, you know, my skillset now is, if you can class it as a skillset, but my knowledge base is there to help.’* (P13/F/ovarian/survivor)

*‘Yes, I have sort of got more active over the years because I see myself being able to give some hope to people when they are talking about their diagnosis … look, you know, here am I seven years on.’* (P11/F/bowel/mal. melanoma/both family and survivor)

Several participants formed close connections with other online community members when they began to communicate with a regular set of like-minded people. One participant memorably described this as becoming part of a ‘tribe’. These groups of people often formed splinter communities based on shared experiences in terms of both personal circumstances and cancer, for instance communities for mothers with cancer, for cancer survivors under the age of 50 years, or for individuals who had undertaken a particular form of treatment (e.g. chemotherapy) in the same year. Furthermore, participants were much more likely to gain status in tribe-like online communities than in communities where individuals were not known to each other. Several participants who were regular community members had been offered roles as moderators of their groups (also referred to as administrators). Moderators were influential members who enforced group rules, removed disruptive group members, and introduced new members to the rest of the community. However, several participants described negative experiences associated with a closer sense of community online. For instance, as individuals became more involved with their online group, they shared and received less support from their offline support network.

*‘There is something about actually finding almost like a tribe… I think you believe rather than hope, hope and sort of believe that they will understand exactly what you are going through and what it feels like. And to a certain point, that reinforces the fact that other people will not be able to have the same understanding.’* (P15/F/breast/thyroid/both family and survivor)

### A journey through different worlds

Participants who engaged with online communities described them as both a distinct social world and a portal to discovering further new online social connections. Online communities had ‘virtual’ qualities which made the interactions online appear distinct from face-to-face interactions. This stemmed from the ability to enter a community and lurk in the background without communicating, thus remaining anonymous or hiding aspects of one’s identity. In addition, many participants explained that discovering communities was like looking into a world that had been hidden from view or *finding a window to a virtual world*. Participants’ perception of this as a ‘social world’ seemed to be compounded by isolation many felt in their role as a person affected by cancer. This description was particularly pertinent to participants at the beginning of their journey with cancer and navigating online communities, as they often felt isolated and emotionally estranged from friends and family as a result of their cancer diagnosis.

*‘It gave her an outlet, a little bit of a window on the world, at least the world she was in. It was very different to the world she used to be in … this was a moderated, err, interaction with the world that she could handle without over tiring her or stressing her out.’* (P14/F/brain/family)

*‘I found an online forum … And then I realised there were other patients out there which of course sound crazy but I didn’t, I didn’t know, you know, I don’t know. I’d heard of malignant melanoma but you don’t know how many people have found they’re suffering from it, you don’t know whether they’re online, you don’t know if they want to communicate.’* (P5/F/melanoma/survivor)

Participants who devoted time to online communities and engaged in conversations online found that they were drawn further into online societies. Most individuals who posted group-wide messages received personal and private invitations to other online communities. These led participants to communities on different websites and platforms. In other words, when participants posted to communities, they were no longer looking into a window; the groups became a door to a social world. Moreover, prolonged interactions created friendships between group members. Thus, many participants felt that they were *being let in to intimate communities*. However, when individuals befriended members in online groups, they were opened up to the possibility of encountering the news of a group member’s cancer progression or the death of a member. Most participants described feeling bereaved after losing an online community member. Similarly, such news reminded participants of their own mortality and participants found this highly distressing.

*‘I think I got talking to somebody on one of the other sites and then we had a private message and then they invited me to join. I did feel a bit like I’d been asked into the sixth form common room [laugh].’* (P17/F/ovarian/survivor)

*‘It is an emotional drain and it, you know, it is awful to read about people suffering. Because, you know, you think well that could be me one day and it is horrible. So that’s why, you know, it’s difficult I suppose.’* (P3/F/mal. melanoma/survivor)

Online communities were perceived as a temporary measure in participants’ lives and necessary whilst they learned how to navigate the cancer experience. Participants who had lived longer with cancer explained that as they adapted to their illness, reading about other members’ experiences could be perceived as superfluous or even distressing. At this juncture, most participants focused on other interests and hobbies or returned to work. Thus, several participants in this study had reached, and most other participants could foresee a time when they would be *moving on from the groups* or no longer participating in online communities.

*‘You’re constantly looking to see what people have put up and what’s going on … constantly looking at the website as well. It’s reinforcing the whole thinking about it and dwelling on it as well. It’s a difficult one … it’s like watching the news about something isn’t it … You know when you’re constantly, constantly looking onto a website and reading it keeps it in your mind.’* (P3/F/mal. melanoma/survivor)

*‘I suspect that as long as my, I think as long as my medical situation is fairly stable and I do not have any additional challenges, then I think I would use them [online communities] less and less.’* (P17/F/ovarian/survivor)

## Discussion

Navigation using online communities was an active, participatory approach to living with cancer. Online communities stimulated individuals’ relationships with cancer, influenced interactions with healthcare professionals, and encouraged active decision-making in cancer care. This was an encouraging finding as an active approach to cancer (sometimes referred to as self-management) has been gaining popularity as an approach to cancer survivorship care in both academic literature and governmental policy [34–36]. Research suggests that self-management can enhance knowledge and skills for self-care, in addition to improving depressed mood, anxiety, and emotional distress [34, 35]. Furthermore, reviews of self-management research have suggested that patient and carer involvement with their own healthcare can improve perceived quality of care, care outcomes, and general population health [36, 37]. Self-management interventions have been an increasing priority in modern healthcare, as individuals managing their own day-to-day care can reduce demand on health service resources [36]. For any health services facing reduced governmental funding, freely available online cancer communities could be a timely self-management resource.

The patient and family benefits of receiving information about cancer have been well documented; information has increased cancer survivor satisfaction [38]; alleviated feelings of uncertainty, loss, and fear; and has allowed people to feel they have increased control over their future [39, 40]. However, studies have consistently found that people affected by cancer have experienced unmet needs for information [41, 42]. The present findings suggested that online communities may meet and support many individuals’ informational needs. This supported previous studies which have suggested that many communities contain high levels of informational support [23, 25, 31]. Moreover, information from online communities was obtained incrementally, in a learning curve over time. This style of information provision contrasted dramatically with the traditional approach of providing large amounts of information in infrequent sittings at healthcare consultations [43, 44]. Evidence has suggested that providing bite-sized chunks of information to individuals over time is a particularly effective form of learning [45, 46]. This could counter cancer survivors’ common complaint of forgetting details of health information and feeling uncertain about the illness [47, 48].

This study found that participants’ personal and social identities could alter as a result of online community interactions. This was a significant finding for cancer populations, as studies have found that many experience identity crises after the diagnosis [49, 50]. Cancer has been considered ‘stigmatising’ because it is associated with progressive illness and dying, and consequently, individuals have struggled to seek support [51, 52]. Moreover, evidence has shown that an identity as a cancer *survivor*, one which emphasises an active involvement in surviving the illness, can promote better quality of life than an identity as a *patient* or *victim* [52, 53]. In the present study, many participants considered their interactions in online communities as evidence that they were personally overcoming, or surviving, challenges they associated with cancer. Thus, online communities could be considered useful tools for helping people to achieve a more positive outlook on their lives with cancer.

This study found that being unique and different to other community members was isolating for cancer survivors. This was consistent with social comparison theories of group behaviour; groups with a strong shared identity could cause individuals to feel excluded if they do not fit the group stereotype [54–56]. In the present study, having a rarer cancer or uncommon treatment path resulted in people stepping off the online cancer navigation journey, preventing them from experiencing social support online. This was concerning because studies have found that a lack of social support can have a direct effect on individuals’ ability to self-manage their personal care [57]. Furthermore, a recent report has suggested that people diagnosed with a rarer cancer had a less positive experience with cancer care and services compared to people diagnosed with the ‘big four’ cancers (breast, lung, bowel, and prostate cancers) [58]. Thus, people affected by rarer cancers may have greater support needs, but online communities may be less able to offer these individuals in-depth support. In order to better understand the support needs of people affected by less common cancers and treatment plans, it may be prudent to conduct further work in this area.

This study found that when online group members died, people affected by cancer became distressed, bereaved, and their identity and perceptions of cancer altered. Studies exploring people’s attitudes to death have found that younger people, in general and in healthcare populations, have a greater anxiety about death and dying than older people [59]. The sample interviewed in the present study was relatively young, and it might be unsurprising that they were concerned about dying. However, the death of friends online was particularly upsetting for participants in this study as it reignited participants’ fears about their own mortality. This was a concerning finding because death anxiety in health populations can be detrimental to psychosocial well-being [60, 61]. Thus, there seemed to be a significant need to support the bereavement experiences of people visiting online cancer communities.

This study demonstrated that lurking online had a valuable function for people affected by cancer. Lurking behaviours have been understudied in cancer communities and in other healthcare communities [27, 62]. Early internet researchers separated lurkers from active participators of online groups, arguing that the former were ‘social freeloaders’ who used online communities for information, with little regard or attention paid to the social environment [27]. Alternatively, the present study supports more recent assertions that lurking is an active and responsive process, in which individuals were listening members of communities [62–64]. Previous online cancer community investigations have discouraged non-posting behaviours [65, 66], arguing that those who did not post messages were not likely to receive social support from online communities. On the contrary, the present study suggested that lurking serves to support a sense of safety, thus increasing individuals’ likelihood of remaining users of online communities and benefiting from the social support offered in group messages. Thus, the findings of this study demonstrated that future high quality online cancer community research should encourage both lurking and posting behaviours.

Participants in this study turned to online communities partly because they were familiar with and habitually used the internet. This seems to suggest that people who are unfamiliar with the internet will not seek out and use online communities. This study did not make direct comparisons between online community users and non-internet using cancer survivors. However, barriers to internet resources may be an important area of future study in order to explore inequality in support provision. A 2015 survey found that a higher proportion of non-internet users were people living with a disability [67]. In addition, whilst population internet access is increasing, rural areas still report lower levels of access than in urban areas [15, 17]. If supportive care increasingly moves online, in accordance with the digital agenda of the European Commission [68], there may be a proportion of people needing but being unable to access support. This may be particularly relevant amongst older adults who use internet technology less frequently than younger adults [15, 17]. Encouraging more of the population to use the internet, particularly amongst demographics with ill health, may provide them with better opportunities for support. However, this study noted that online community use posed challenges for individuals, particularly when deciphering the relevance of the medical content of messages. Therefore, as we encourage individuals to use online health support, it would be prudent to simultaneously increase their ability to evaluate online information. This could be delivered by incorporating critical appraisal tools for online information with online cancer community resources. Additionally, there is a precedence for providing digital training in western countries; for instance, digital inclusion and literacy training is advocated as a strategic objective of the European Union [69]. Thus, skills in appraising online health information could be included in digital literacy training to strengthen individuals’ ability to positively navigate through health communities.

As a cross-sectional qualitative approach, it was unsurprising that this study obtained a limited range of demographics represented by participants. The samples were mostly white and female, which is a common limitation in western health research [70]. In addition, the sample of participants was relatively young for a population of people affected by cancer. There may have been several reasons for this finding. For instance, younger populations have been more responsive and capable in terms of technology and internet communication [15–17]. Indeed, a mediating factor for participants using online communities was their familiarity with the internet. Thus, there may have been a greater number of younger populations in online communities and willing to participate in this study. In addition, there were a surprising number of requests to participate from people affected by melanoma and ovarian cancer. Melanoma is the fifth most common cancer in the UK, whilst ovarian cancer is the 15th most common cancer in the UK [71]. These cancers were significantly less common than the four most diagnosed cancers in the UK (breast, lung, prostate, and bowel), which together account for approximately 53% of UK cancer diagnoses [71]. The prevalence of people affected by melanoma was interesting and could suggest that communities were particularly utilised by groups of people affected by melanoma and ovarian cancer. Alternatively, the prevalence of people affected by melanoma in this study could have been caused by the online recruitment strategy. Several participants contacted LJH from the same private online community for melanoma, and it emerged that an advertisement had been shared in this group. This suggested that online recruitment can lead to an incidental snowballing sampling strategy.

The aim of this study was to generate a theory of online cancer community use from the experiences of people affected by cancer. By utilising constructivist grounded theory, unique interactions with online communities, such as lurking and seeking support from online networks, were explored in depth. Thus, we were able to develop a *substantive* theory of behaviours with online cancer communities [33, 72]. Hence, the theory has been situated in the experiences of cancer survivors, but inferences can be made across health conditions with similar characteristics and experiences with other online support services. Online communities are used for a range of illnesses including life-threatening conditions, such as HIV/AIDS, and chronic conditions, such as diabetes, fibromyalgia, and depression [73]. We can find no other theories that describe how online health communities shape the experience of illnesses. Cancer is an umbrella term for a wide-ranging set of experiences which can be experienced as both a life-threatening and chronic illness. This study attempted to capture this diversity by sampling from individuals affected by a range of different cancer diagnoses and by including families. Therefore, whilst this theory is situated in the framework of cancer survivorship, it offers an interpretation of how online communities can impact other disease groups, as it highlights the multifaceted influence of online communities.

## Conclusions

The paper has offered a detailed and nuanced interpretation of how and why communities have been valuable for supporting peoples’ journeys with cancer. Before a policy can advocate the use of particular online communities, there must be reliable evidence to show that the communities will benefit people affected by cancer [74]. This qualitative study has provided a testable theory for further quantitative investigations to conclusively determine the psychosocial benefits of online cancer community use for cancer survivorship. Moreover, this study has highlighted several practical ways that cancer survivorship can be improved through online community engagement. Firstly, online communities could be used to support existing programmes of cancer self-management, particularly for individuals who require a positive identity in survivorship, and a source of experiential information. Secondly, this study demonstrated that people affected by rarer cancers should be warned about the potential for isolation online, and offered additional psycho-social support. Thirdly, lurking and posting behaviours can both be undertaken for individuals wishing to use online communities to navigate cancer. Fourthly, experiences in online communities could be supported by increasing individuals’ digital literacy when evaluating shared cancer information and by providing support for bereavement online.

## Appendix

**Table 2:** Interview schedule

Tell me about using online groups for cancer
What drew you to online groups/communities?
Can you tell me a little about reading/posting messages in online communities?
Have you found anything about online support groups that are helpful?
Have you found anything about online support groups to be unhelpful?
How important have the groups been to you?
